# Navigating Calcium and Reactive Oxygen Species by Natural Flavones for the Treatment of Heart Failure

**DOI:** 10.3389/fphar.2021.718496

**Published:** 2021-11-09

**Authors:** Tianhao Yu, Danhua Huang, Haokun Wu, Haibin Chen, Sen Chen, Qingbin Cui

**Affiliations:** ^1^ Department of Cardiology, Guangdong Second Provincial General Hospital, Guangzhou, China; ^2^ School of Public Health, Guangzhou Medical University, Guangzhou, China

**Keywords:** heart failure, calcium overload, ROS, flavones, treatment

## Abstract

Heart failure (HF), the leading cause of death among men and women world-wide, causes great health and economic burdens. HF can be triggered by many factors, such as coronary artery disease, heart attack, cardiomyopathy, hypertension, obesity, etc., all of which have close relations with calcium signal and the level of reactive oxygen species (ROS). Calcium is an essential second messenger in signaling pathways, playing a pivotal role in regulating the life and death of cardiomyocytes via the calcium-apoptosis link mediated by the cellular level of calcium. Meanwhile, calcium can also control the rate of energy production in mitochondria that are the major resources of ROS whose overproduction can lead to cell death. More importantly, there are bidirectional interactions between calcium and ROS, and such interactions may have therapeutic implications in treating HF through finely tuning the balance between these two by certain drugs. Many naturally derived products, e.g., flavones and isoflavones, have been shown to possess activities in regulating calcium and ROS simultaneously, thereby leading to a balanced microenvironment in heart tissues to exert therapeutic efficacies in HF. In this mini review, we aimed to provide an updated knowledge of the interplay between calcium and ROS in the development of HF. In addition, we summarized the recent studies (*in vitro*, *in vivo* and in clinical trials) using natural isolated flavones and isoflavones in treating HF. Critical challenges are also discussed. The information collected may help to evoke multidisciplinary efforts in developing novel agents for the potential prevention and treatment of HF.

## Introduction

Heart diseases, including cardiovascular diseases, the world’s leading cause of death, are composed with a class of chronic, progressive, and/or lethal diseases, such as high blood pressure, high blood cholesterol, abnormal heart rhythms, coronary artery disease, ischemic heart disease, stroke, heart attacks, etc. (Virani et al., 2021). Heart failure (HF), the late stage of heart diseases, is a condition in which there is a dramatically reduced supply of blood pumped by the muscle of the heart (Dryer et al., 2021). While HF is a chronic and progressive disease, its onset and consequence are acute and prominent (Weintraub et al., 2010). In China, there are approximately 14 million patients suffering from HF, with a prevalence rate of 1.3% (0.9% in 2000). As in the United States, approximately 1.5 million people experience HF every year, causing over 690,000 deaths in 2019 (Ahmad and Anderson, 2021), indicating a great burden to health and finance.

While varied therapeutics for heart diseases that may lead to HF are available, such as nitrates, calcium channel blockers, angiotensin-converting enzyme (ACE) inhibitors, angiotensin II receptor blockers, and 3-hydroxy-3-methylglutaryl coenzyme A (HMG-CoA) reductase inhibitors, etc., novel agents, including those effective in preventing HF, are in urgent need to reduce the high morbidity, and mortality. Growing evidence has suggested that the prevention of HF is quite imperative (Horwich and Fonarow, 2017; Wang, 2019). Among all the newly developed regimens, nature-derived products can exert huge potential because of their unique properties and multiple functions such as regulating calcium and reactive oxygen species (ROS) levels in cells.

Here, we attempted to summarize the interactions between calcium cation (Ca^2+^) signaling and ROS level, both of which contribute to the progression of heart disease as well as HF (Bertero and Maack, 2018). Calcium can work as a direct signaling transductor or a second messenger in regulating neuronal transmission, electrical excitation and contractile function of myocytes (Landstrom et al., 2017; Terrar, 2020), or in promoting the growth, life or death of cells such as proliferation and apoptosis (Rizzuto et al., 2003; Lemos and Ehrlich, 2018) which has an intimate connection with the level of cellular ROS (Orrenius et al., 2003). Meanwhile, similar to high levels of ROS, high levels of cytoplasmic calcium, a term called calcium overload, can also induce cell death (Zhivotovsky and Orrenius, 2011). Therefore, it is feasible that the close interaction between ROS and calcium in inducing cell death can be endowed with therapeutic implications (Münzel et al., 2017). Interestingly, there are many natural products that can simultaneously reduce calcium overload and ROS over-production, exerting cardiovascular protective, and HF-preventing effects (Jiang et al., 2016; Mohiuddin, 2019). Flavones including isoflavones, which are one of the most abundant components in plants and fruits, have been intensely studied and applied in markets as a dietary supplement to prevent the incidence of heart diseases including HF (Dixon and Pasinetti, 2010; McCullough et al., 2012; Zamora-Ros et al., 2013). Therefore, we also attempted to summarize the current status (those studies conducted in the past decade) and challenges in using flavones as therapeutic agents in HF via the dual-regulation of calcium and ROS. The information gained may serve as a foundation for further in-depth study, including pharmacological and chemical modification research, and the development of flavones in clinical use.

## The Interplay Between Calcium and ROS in Inducing Cardiomyocytes Death

ROS over-production and calcium accumulation in acute myocardial ischemic injury can be attributed to be the major causes of damage to the heart (Shen and Jennings, 1972). Calcium plays key roles in multiple aspects of heart tissue and cell biology. In this review, we highlighted its role in inducing cell death. Calcium concentrations in the outside and inside of cells, in endoplasmic reticulum (ER), and mitochondria are pivotal for maintaining cell functions, and its alterations could lead to cell death (Bagur and Hajnóczky, 2017). Calcium overload, especially in the mitochondria of cardiomyocytes, can cause HF as shown in cell-based models and mouse models (Luo and Anderson, 2013; Santulli et al., 2015; Mora et al., 2018). The malfunctional mitochondria due to calcium overload can further produce more ROS, which may also finally contribute to HF (Luo and Anderson, 2013; Santulli et al., 2015; Bertero and Maack, 2018).

ROS are one of the main inducers of cell death (Ryter et al., 2007). Normally, the ROS level remains in a controllable condition mediated by the producing systems and the active antioxidant enzymes (eliminating systems) in cells, and they, when working as signal transductors, can closely participate in almost every aspect of cell biology (Cui et al., 2018a; Bock and Tait, 2020). Under stress and malfunctioning conditions, the ROS level in cells can be increased due to varied reasons, leading to the apoptosis initiation (Cui et al., 2018a). Oxidative stress due to over-produced ROS is one of the hallmarks of cardiovascular disease, which has close connections with the progression of ischemia-reperfusion damage and atherosclerosis, both of which can eventually lead to HF (Panth et al., 2016).

**FIGURE 1:**
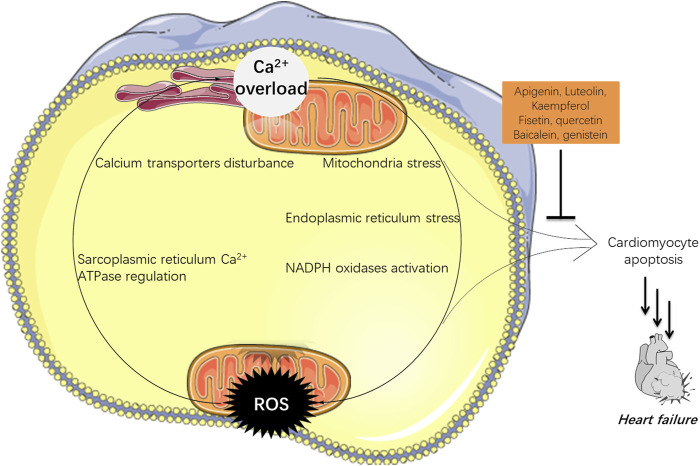
The interactions between calcium and ROS in HF. Over-loaded calcium could not only induce apoptosis, but also stimulate the production of ROS which may initiate cell death. Flavones hold potential in navigating both of them which can be adopted in HF treatment.

There are bidirectional interactions between calcium and ROS as shown in Figure 1 (Görlach et al., 2015). Briefly, calcium can modulate the formation and production of ROS. First, ER and mitochondria are two major producers of various ROS; and calcium can induce ER stress, and enhance ATP production in mitochondria that requires oxygen, resulting in over-produced ROS. Second, NADPH oxidases (NOXs) that are calcium dependent, are another major source of ROS such as H_2_O_2_ and O_2_^−^ (Rastogi et al., 2016; Burtenshaw et al., 2017). Calcium can either activate NOXs *via* directly binding to NOXs at certain domains or in an indirect way through signal transduction, leading to ROS over-production (Bánfi et al., 2004). Endothelial nitric oxide synthase (eNOS), one of three isoforms that synthesize nitric oxide (NO) (Cui et al., 2019), is another enzyme that is calcium dependent (Aoyagi et al., 2003; Devika and Jaffar Ali, 2013). Calcium can activate calmodulin, which then binds to eNOS, leading to its efficient NO production (Sessa, 2004). Furthermore, calcium can also induce ROS generation by impacting other key ROS-maintaining enzymes such as voltage dependent anion channels (VDAC) (Feno et al., 20192019), or certain complexes form the electron transporting chain (ETC) located in the inner mitochondrial membrane (Adam-Vizi and Starkov, 2010), etc.

Meanwhile, ROS can also negatively influence myocardial calcium handling, causing arrhythmia, and augmenting cardiac remodeling by inducing hypertrophic signaling and apoptosis, which later contributes to HF (Senoner and Dichtl, 2019). ROS are ready to attack cellular biomolecules including calcium transporters on cell membranes or organelles’ membranes including ER and mitochondria, therefore affecting the calcium homeostasis (Zimmerman et al., 2011). Free radical H_2_O_2_ can bind to the residue of Cys674 at the sarcoplasmic reticulum Ca^2+^ ATPase (SERCA), leading to disturbed cardiac myocyte in a rat heart (Qin et al., 2013).

In a word, high levels of ROS can increase the uptake of calcium in cells; meanwhile the calcium level in cells can also stimulate the production of ROS. These two events working together can finally induce cardiomyocyte death and eventually HF.

## Natural Flavones Exhibit Potential in Treating HF via Dual Regulation of Calcium and ROS

**FIGURE 2:**
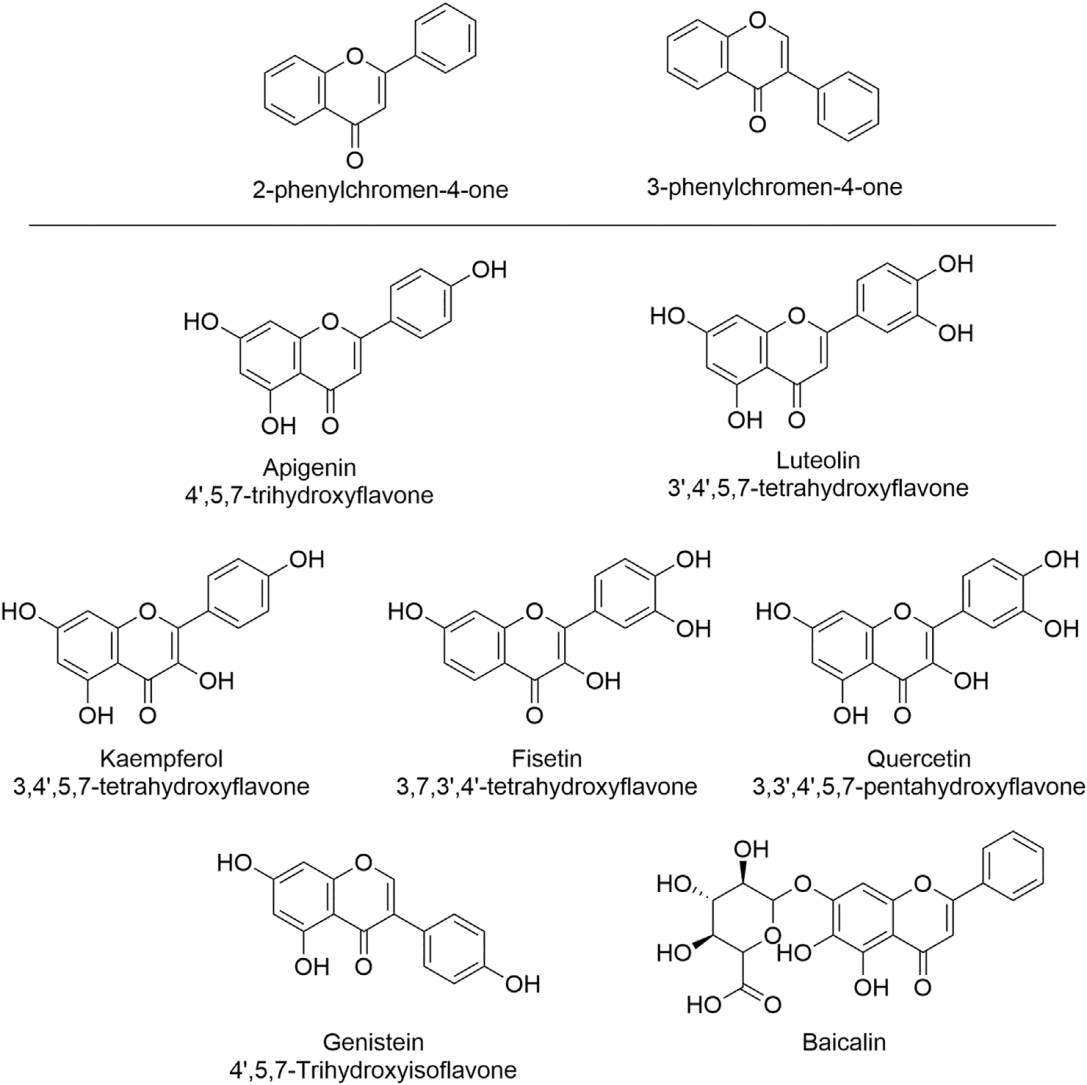
The structures of flavones and isoflavone that show HF-treating/preventing effects *via* the regulation of both ROS and calcium signal. These natural products can serve as leading compounds that can undergo structural modification to achieve the selective regulation of ROS and calcium.

Flavones, including isoflavones, are a class of natural products categorized as flavonoids, sharing a common backbone of 2-phenylchromen-4-one (flavone) or 3-phenylchromen-4-one (isoflavone) (Figure 2) (Hostetler et al., 2017). Natural flavones are rich in fruits, vegetables, soybean, herbal plants, honey, and they have been used as herbal medicines for over 1,000 years. Importantly, the isolated/purified components have been used as supplemental nutrients for decades (Singh et al., 2014). Currently, dozens of flavones are under clinical trials for the treatment of diseases associated with cardiovascular dysfunction and other diseases including neurodegenerative diseases, diabetes mellitus, cancers, etc., suggesting their huge potentials (Hostetler et al., 2017; Cui et al., 2018b). Flavones are known as multi-targeting or multi-functional compounds since they can regulate/target multiple enzymes *in vivo* (Qiu et al., 2018; Ye et al., 2019), such as silent mating type information regulation 2 homolog (SIRT) (Kang et al., 2018), ABC transporters (Li and Paxton, 2013), cyclin-dependent kinases (CDKs) (Khuntawee et al., 2012), and certain microRNA (Lin et al., 2018), etc. In addition to their multi-functional property, flavones also exert medical efficacies *via* multi-mechanisms including the regulation of both ROS and calcium that contribute significantly to HF. A retrospective clinical meta-analysis of 23 years among 56,048 Danish people has indicated that the consumption of certain flavonoids (500 mg/day) can reduce the incidence and mortality of cardiovascular diseases (Bondonno et al., 2019), and such efficacies have been validated by other studies as well (Ponzo et al., 2015; Dalgaard et al., 2019), suggesting the beneficial effects of flavonoids in treating HF. Indeed, growing *in vitro* and *in vivo* studies have proven such effects (Mozaffarian and Wu, 2018). Here, we focus on those flavones and isoflavones (Figure 2) that exert their heart protective effects *via* dual regulating ROS and calcium signal.

Apigenin (4′,5,7-trihydroxyflavone), a dietary supplement that has demonstrated the ability to regulate both ROS and calcium (Maher and Hanneken, 2005; Wu et al., 2021), suggesting its potential in treating HF. Li et al. (2017) reported that apigenin (50 mg/kg) could relieve myocardial injury induced by endotoxin and decrease the death rate of cardiomyocytes in mice, suggesting a cardioprotective effect (Li et al., 2017). Apigenin worked *via* reducing oxidative stress as confirmed by increased cardiac glutathione (GSH) level, oxidative stress markers, and pro-inflammatory cytokines including tumor necrosis factor (TNF-α), interleukin 1β (IL-1β), macrophage inflammatory protein-2 (MIP-2) which have intimate networks with Ca^2+^-associated signals (Hendy and Canaff, 2016; Li et al., 2017).

Luteolin (3′,4′,5,7-tetrahydroxyflavone) is a flavone that has been serving as a supplemental nutrient for decades for improving memory and brain health (Swaminathan et al., 2019; Wang et al., 2021). Luteolin can protect heart from damage caused by over-produced ROS and over-loaded calcium (Wang et al., 2012; Yan et al., 2018). Wang et al. (2012) found that in an animal model of type I diabetic cardiomyopathy, luteolin (200 mg/kg) maintained certain cardiac functions as measured by the left ventricular systolic pressure, left ventricular developed pressure, left ventricular end diastolic pressure, and maximal rate of rise/fall left ventricle pressure development (Wang et al., 2012). They also found that luteolin worked by reducing oxidative stress as confirmed by decreased ROS-producing proteins and signal pathways (Wang et al., 2012). Madhesh and Vaiyapuri (2005 and 2012) found that luteolin (0.3 mg/kg/day) could protect cardiac function and prevent myocardial infarction by reducing mitochondrial lipid peroxidation (a route that produce ROS *in vivo*) and increasing the mitochondrial antioxidant levels as shown in isoproterenol induced myocardial infarction model in rats (Manju et al., 2005; Madhesh and Vaiyapuri, 2012). Luteolin appears to be an inhibitor of L-type calcium channels as confirmed by Yan et al. (2018), Yan et al. (2019). Luteolin (7.5, 15, or 30 µM) ameliorated calcium overload in freshly isolated cardiomyocytes, accompanied by suppressed Protein Kinase A (PKA) activity and enhanced Ca^2+^-Mg^2+^-ATPase activity (Yan et al., 2018). Luteolin’s regulatory role in calcium was also confirmed by Li et al.’s studies in 2015 and 2017 (Nai et al., 2015; Hu et al., 2017a).

Kaempferol is another widely used dietary supplement with a chemical name of 3,4′,5,7-tetrahydroxyflavone, and it can protect heart *via* the navigation of both ROS and calcium (An and Kim, 2015; Guo et al., 2015). An *in vitro* study by Guo et al. (2015) showed that in A/R-induced injury model, kaempferol (10, 20, or 40 μM) inhibited mitochondria-mediated apoptosis and increased the cell viability of cardiomyocytes *via* reducing ROS production mediated by activating SIRT1 (Guo et al., 2015). Calmodulin kinase II (CaMKII), a key player in calcium signaling pathways, can be activated by higher levels of ROS, thereby resulting in abnormally slow heart rhythm or cardiomyocytes death (Di Carlo et al., 2014; Santalla et al., 2014). Kaempferol (15 mM) significantly reduced the CaMKII oxidization and sinus nodal cell death, warranting further *in vivo* verification (An and Kim, 2015). It is also worth noting that the concentration used in this study is 15 mM, which is much higher than the other studies that fall in submicromolar concentrations. Such high concentrations may cause problems in clinical trials when translating the *in vitro* doses into those in humans, requiring an in-depth pharmacokinetic study.

Fisetin (3,7,3′,4′-tetrahydroxyflavone) is an abundant flavone existing in strawberry, apple, persimmon, grape, onion, and cucumber. Fisetin demonstrated multiple health benefits including preventing HF via regulating ROS and calcium (Rodius et al., 2020). Shanmugam et al. (2018) confirmed the cardiovascular protective effect of fisetin using a Langendorff isolated heart perfusion system (Shanmugam et al., 2018). Fisetin pretreatment (20 mg/kg) showed a strong protective effect against the damage induced by myocardial ischemia reperfusion in the isolated rat heart. Fisetin improved mitochondrial physiology, biogenesis, and functions including maintaining the ETC and reducing superoxide (O_2_^−^) generated in mitochondria, suggesting a mitochondria-mediated mechanism (Shanmugam et al., 2018). The further *in silico* analysis and computer-aided docking study showed that fisetin might be a potent glycogen synthase kinase 3β (GSK3β) inhibitor (Shanmugam et al., 2018), warranting further study.

Quercetin (3,3′,4′,5,7-pentahydroxyflavone) is an important dietary flavone in fruits and vegetables, and it possesses anti-inflammatory and anti-oxidative properties which may benefit the patients with cardiovascular diseases (Patel et al., 2018). Jing et al. (2016) found that quercetin pretreatment (20 mg/kg) reversed cardiomyocytes apoptosis induced by posttraumatic stress and it restored cardiac function as shown in a rat model (Jing et al., 2016). *In vitro* study of pretreatment with quercetin at 20 μM showed that it can obviously maintain the cell viability, decreased TNF-α, ROS level and calcium overload in H9c2 cells, suggesting the beneficial effects of quercetin in treating cardiac injury (Jing et al., 2016). Quercetin can also protect the heart from myocardial ischemia reperfusion injury via the dual regulation of ROS level and calcium overload (Zhang et al., 2020). Furthermore, a meta-analysis of clinical trials showed that the consumption of quercetin (at the dose of more than 500 mg/day) exhibited significant reduction of blood pressure, suggesting a beneficial effect of quercetin in HF (Serban et al., 2016).

Baicalein, enriched in natural products and herbal medicines, is a glycosylated flavone that regulates ROS and calcium in cells (Xin et al., 2020), showing promising therapeutic effects in treating and preventing HF (Zhao et al., 2016). Zhao et al. (2016) found that in HF *in vivo* model established by abdominal aorta constriction in rats and *in vitro* isoproterenol-induced H9C2 cells, baicalein (50, 100, and 200 mg/kg *in vivo* or 5, 10, 20 μM *in vitro*) significantly alleviated HF syndromes by improving heart function as confirmed by hematoxylin-eosin and ELISA measuring the pathomorphological changes and down-regulated TNF-α, angiotensin II, and BNP in peripheral blood (Zhao et al., 2016). Baicalein reduced myocardial fibrosis *in vivo* through inhibiting the expression and activities of matrix metalloproteinase-2 and -9 (MMP-2/9). Furthermore, baicalein was found to suppress isoproterenol-induced cardiomyocytes hypertrophy and apoptosis *in vivo* and *in vitro*, probably via regulating calcium related proteins such as the phosphorylated Ca^2+^/calmodulin-dependent protein kinase II (CaMKII), Na^+^/Ca^2+^-exchangers (NCX1) and sarcoplasmic reticulum Ca^2+^ ATPase 2 (SERCA2) (Zhao et al., 2016).

Genistein (4′,5,7-trihydroxyisoflavone) is an isoflavone that is found in soy-based products, being widely used as a supplemental nutrient for years (Williamson-Hughes et al., 2006; Mamagkaki et al., 2021). Genistein is also a dual regulator of ROS and calcium (Uddin and Kabir, 2019). Matori et al. (2012) found that genistein (1 mg/kg/day for 9 days) could restore cardiopulmonary structure and function, and reverse the loss of capillaries induced by pulmonary hypertension in the rat model, demonstrating its potential in preventing HF (Matori et al., 2012). In addition, a randomized double-blind case-control study conducted among postmenopausal women with metabolic syndrome showed that genistein (54 mg/day) significantly improved heart functions measured by the left ventricular ejection fraction and remodeling, suggesting a favorable outcome when applied in human with cardiovascular diseases (De Gregorio et al., 2017).

Other potential flavones such as rutin, quercetin-3-O-rutinoside which is the glycosylated quercetin (Chu et al., 2014; Lv et al., 2018), chrysin (5,7-Dihydroxyflavone) (Farkhondeh et al., 2019; Xingyue et al., 2021), wogonin (5,7-Dihydroxy-8-methoxyflavone) (Khan et al., 2016; Khan and Kamal, 2019), also possess cardiovascular protective and HF-preventing efficacies *via* an ROS-calcium associated mechanism, rendering them as attractive drug candidates or dietary supplements.

## Discussion and Future Perspective

The information discussed above has indicated that 1) there is a vicious cycle between overloaded calcium and over-produced ROS, and both contribute to HF; 2) certain flavones can protect cardiovascular *via* down-regulating both intracellular calcium content and ROS level, thereby demonstrating potentials in preventing/treating HF as summarized in Table 1. As shown in the original studies, luteolin, kaempferol, and baicalein, demonstrated a dose-dependent mode of action; while the other four including apigenin, fisetin, quercetin, and genistein, were tested with one dose/concentration to exert the HF-treating/preventing effects, warranting further pharmacological study *in vivo*. In addition, combinational strategies of certain flavones and conventional drugs can also be developed and applied in HF treatment via synergistic effects (Guerrero et al., 2012; Zeka et al., 2017).

**TABLE 1:** Summary of the discussed flavones.

Flavones	Experimental model	Doses/Effects	Mechanisms	References
Apigenin	*In vivo* myocardial injury	Relieving myocardial injury at 50 mg/kg	Reducing ROS and negatively regulating calcium-related signal	Li et al. (2017)
Luteolin	*In vivo* type I diabetic cardiomyopathy	Maintaining cardiac functions at 200 mg/kg	Reducing oxidative stress	Yan et al. (2018)
	*In vivo* myocardial infarction model	Protecting cardiac function at 0.3 mg/kg/day	Reducing mitochondrial lipid peroxidation	Manju et al. (2005), Madhesh and Vaiyapuri (2012)
Kaempferol	*In vitro* anoxia/reoxygenation induced injury model	Inhibiting apoptosis at 10, 20, or 40 μM	Reducing ROS production mediated by activating SIRT1	Guo et al. (2015)
	Isolated Langendorff heart	Protecting sinus node	Reducing CaMKII oxidization	An and Kim (2015)
Fisetin	The Langendorff isolated heart perfusion system	Protective effect against myocardial ischemia reperfusion at 20mg/kg	Decreasing ROS and calcium	Shanmugam et al. (2018)
Quercetin	*In vivo* posttraumatic cardiac injury model	Preventing apoptosis and cardiac dysfunction at 20 mg/kg	Decreasing ROS and calcium	Jing et al. (2016)
	*In vitro* H9c2 cardiomyoblasts	Maintaining cell viability at 20 μM	Decreasing ROS and calcium	Jing et al. (2016)
Baicalein	*In vivo* HF model and *In vitro* in H9C2 cells	Alleviating HF syndromes and reducing myocardial fibrosis at 50, 100, and 200 mg/kg and inhibiting apoptosis at 5–20 μM	Inhibiting MMP-2/9, reducing ROS, and regulating calcium signal	Zhao et al. (2016)
Genistein	*In vivo* pulmonary hypertension model	restore the structure and function of heart and lung at 1 mg/kg/day for 9 days	Decreasing ROS and calcium	Matori et al. (2012)

Meanwhile, cautions should also be made. Firstly, these flavones are not specific regulators of ROS or calcium, undermining their potential as drug candidates which require the selective targeting of certain pathogenic mechanisms/proteins. As for these small-molecule flavones, it appears to be true that none of them has a selective bio-target *in vivo*, and it is well accepted that most of them might exert their bioactivities *via* interacting with membrane proteins (Cyboran et al., 2012; Ingólfsson et al., 2014; Phan et al., 2014), requiring more studies such as medicinal chemical modification to improve the selectivity and druglikeness (Boniface and Elizabeth, 2019). As far as the authors concerned, it seems to be more reasonable to develop them as supplemental nutrients in preventing HF.

Secondly, the dual-regulation of ROS and calcium might not be the mere mechanism that leads to cardio-protective effects by flavones (Najjar and Feresin, 2021). HF, the late stage of heart diseases, can be triggered by various factors; consequently, flavones can also exert HF-preventing efficacies *via* multiple mechanisms which have been intensively studied over the past decade (Grassi et al., 2013; Choy et al., 2019; Ciumărnean et al., 2020a; Ciumărnean et al., 2020b; Fusi et al., 2020; Yamagata and Yamori, 2020; Jiang et al., 2021). This fact can further support the strategy of developing flavones as supplemental nutrients.

Last, in spite of the fact that the aforementioned flavones can generally reduce the level of ROS in cardiomyocytes, a significant proportion of them (at varied concentrations) can also induce the production, leading to cell death which can be applied in cancer treatment (Lu et al., 2007; Lin et al., 2011; Shih et al., 2017; Souza et al., 20172017; Cui et al., 2018b; Cataneo et al., 2019; Korga et al., 2019). Thus, the therapeutic windows of each flavone should be determined before their application (or trials) in humans.

Multiple clinical trials are ongoing and several conducted previously have been completed as shown in the Supplemental Table S1. It is worth noting that quercetin, whose name has been used since 1857, has been widely tested in clinical trials for the treatment of different diseases including heart diseases. As one of the most abundant, and widely studied and applied as nutritional supplement (Jing et al., 2016; Patel et al., 2018), it is the authors’ opinion that quercetin has a greater potential in treating/preventing HF among all the others. However, by far, using flavones as drug candidates in HF treatment/prevention is still in its early stage. One of the major obstacles that refrain the effects *in vivo* and in clinical trials is that the stability, selectivity, and overall poor bioavailability that fails to reach consistent exposure levels, etc. (Ross and Kasum, 2002; Wu et al., 2011; Thilakarathna and Rupasinghe, 2013; Hu et al., 2017b). Bioavailability of certain flavones has been tested in human, and the results indicated that only a small proportion can be absorbed (Meyer et al., 2006; Kanaze et al., 2007), such as15–24% of genistein (Lu and Anderson, 1998). Such low bioavailability may require high doses in humans, and a typical dose is 500 mg/day, and doses below this may not benefit patients with heart diseases/conditions (Kirienko and Radak, 2016; Serban et al., 2016; Bondonno et al., 2019). Therefore, to achieve the full potential in HF, further *in vitro* and *in vivo* studies are required to determine the dose, administration methods, safety, and pharmacokinetic and pharmacodynamics profiles.

## Conclusion

Overload of calcium and elevated ROS production can form a vicious cycle to induce cardiomyocytes death that may finally lead to HF. A number of flavones show the dual-regulation of calcium and ROS, demonstrating their therapeutic potential in HF.
