# Supplementary material for: Navigating Calcium and Reactive Oxygen Species by Natural Flavones for the Treatment of Heart Failure
Source: Front Pharmacol. 2021 Nov 9;12:718496. doi: 10.3389/fphar.2021.718496 (PMC8630744; doi:10.3389/fphar.2021.718496)
Supplement: Supplementary file 1 [file Table1.pdf]

Supplemental Table

A mini review of navigating calcium and reactive oxygen species by natural flavonoids and their potentials to treat heart failure

Tianhao Yu[1,#,*], Danhua Huang[2,#], Haokun Wu[1], Haibin Chen[1], Sen Chen[1], Qingbin Cui[2]

[1] Department of Cardiology, Guangdong Second Provincial General Hospital, Guangzhou, Guangdong 510314, China. [2] School of Public Health, Guangzhou Medical University, Guangzhou, Guangdong 511436, China.

[#] Tianhao Yu and Danhua Huang contributed equally.

** Corresponding author: Tianhao Yu, email: eshldr@126.com*

Table 1. The clinical trials using flavonoids for the treatment of HF-related diseases

**Drug candidates**	**Indications/Condition**	**Results**	**ClinicalTrials.gov Identifier/Refs**
Apigenin/luteonin	High cholesterol	Not revealed yet	NCT04114916
Naringenin	Mild hypercholesterolemia	Not revealed yet	NCT00539916
	Mild hypercholesterolemia	No effects on serum cholesterol	(67)
Kaempferol	Dyslipidemia	Not revealed yet	NCT04110392
Quercetin	Hypertension	Reducing blood pressure	NCT01691404, (87)
	Coronary artery disease	Not revealed yet	NCT03943459
Genistein	Metabolic syndrome	Improving cardiac function	NCT00541710

67. Demonty I., Lin Y., Zebregs Y. E., Vermeer M. A., van der Knaap H. C., Jakel M. and Trautwein E. A.: The citrus flavonoids hesperidin and naringin do not affect serum cholesterol in moderately hypercholesterolemic men and women. *J. Nutr.* (2010) 140(9): 1615-20.

87. Serban M. C., Sahebkar A., Zanchetti A., Mikhailidis D. P., Howard G., Antal D., Andrica F., Ahmed A., Aronow W. S., Muntner P., Lip G. Y., Graham I., Wong N., Rysz J. and Banach M.: Effects of Quercetin on Blood Pressure: A Systematic Review and Meta-Analysis of Randomized Controlled Trials. *J. Am. Heart Assoc.* (2016) 5(7)
